# Spectroscopic and Pulse Radiolysis Studies of Water–Ethanolic Solutions of Albumins: Insight into Serum Albumin Aggregation

**DOI:** 10.3390/ijms26136283

**Published:** 2025-06-29

**Authors:** Karolina Radomska, Marian Wolszczak

**Affiliations:** 1 Institute of Applied Radiation Chemistry, Faculty of Chemistry, Lodz University of Technology, 93-590 Lodz, Poland; karolina.radomska@p.lodz.pl; 2 Centre of Papermaking and Printing, Lodz University of Technology, Wolczanska 221, 93-005 Lodz, Poland

**Keywords:** albumins, ethanol, ethanolic solutions, pulse radiolysis, aggregation, reduction in albumins

## Abstract

Albumin-based nanoparticles are promising drug delivery systems due to their biocompatibility, biodegradability, and ability to improve targeted drug release. Among various preparation methods, radiation-induced cross-linking in the presence of ethanol has been proposed in the literature as an effective method for producing protein nanoparticles with preserved bioactivity and controlled size. However, the mechanisms by which ethanol radicals contribute to protein aggregation remain insufficiently understood. In this study, we investigate the role of ethanol in the aggregation of albumins to determine whether its presence is necessary or beneficial for nanoparticle formation. Using pulse radiolysis, spectroscopy methods, resonance light scattering (RLS), and near-infrared (NIR) spectroscopy, we examined aqueous ethanol solutions of albumins before and after irradiation. Our results show that ethanol concentrations above 40% (*v*/*v*) significantly promote both radiation-induced and spontaneous protein aggregation. Mechanistic analysis indicates that ethanol radicals react with albumin similarly to hydrated electrons, mainly targeting disulfide bridges. This reaction leads to the formation of sulfur-centered radicals and the formation of intermolecular disulfide bonds that stabilize protein nanostructures by excluding the formation of dityrosine bridges, as described in the literature. In contrast, ethanol concentration below 40% does not favor the radiation-induced aggregation compared to the solution containing t-BuOH. These results provide novel insights into the role of organic cosolvents in protein aggregation and contribute to a broader understanding of the mechanisms of formation of albumin-based nanoparticles using ionizing radiation.

## 1. Introduction

Currently, albumin nanoparticles (AN) are of great interest as a key system in personalized medicine, theranostics, and precision medicine [1,2]. Their use in medicine mainly concerns drug delivery systems and is recommended due to their unique properties: non-toxic, non-antigenic, and biodegradable. An interesting application of protein aggregates is hydrogels with a microsphere structure, which use bovine serum albumin (BSA) as a dielectric layer for capacitive pressure sensors. This type of BSA sensor can be used to monitor a variety of health data, including finger and wrist flexion and heart rate [3]. Moreover, protein hydrogels are also suitable for applications such as artificial electronic skin and wearable devices.

There are at least six strategies for synthesizing albumin nanoparticles [1]. ANs are prepared through methods such as desolvation, thermal gelation, emulsification, NAB technology (nanoparticles albumin-bound technology), self-assembly, and nanospray drying [1]. More than forty years ago, it was first described that the irradiation of an albumin solution (X-rays) under anaerobic conditions (N_2_-saturated BSA solution) causes the formation of covalent bonds between protein molecules [4]. This inspired us to investigate the effect of ionizing radiation on the HSA structure under reductive conditions and the possibility of its application in the synthesis of protein aggregates [5]. In the desolvation method, the addition of an appropriate amount of a desolvating agent (non-solvent), such as ethanol, reduces the degree of hydration of the albumin surface and leads to the formation of nanoparticles in an aqueous solution. Such spontaneously formed albumin nanoparticles are unstable. The cross-linking process improves the quality of the ANs, increasing their stability and durability. The addition of a chemical agent (e.g., glutaraldehyde) to the albumin coacervate leads to stabilization of AN. Albumin nanoparticles prepared using this method must be subjected to the process of removing the cross-linking agent. In this publication, we describe the process of AN formation without the use of a chemical reducing agent, replacing it with the use of ionizing radiation under reducing stress conditions (hydrated electron and 1-hydroxyethyl radical). At the end of the last century, it was shown that the radiation-induced intermolecular cross-linking method, first developed by Ulański and Rosiak, is a very suitable method for the synthesis of protein nanoparticles [6].

The possibility of effective use of ionizing radiation to obtain protein nanoparticles under various conditions has been demonstrated by many different research groups [7]. Ethanol (EtOH)–water solutions of BSA were exposed to γ radiation (dose 10 kGy), resulting in nanostructures with controlled sizes ranging from 20 to 40 nm, depending on the ethanol concentration [7]. Recently, the formation of papain nanostructures has been described by Varca et al. in detail [8,9,10]. They irradiated a papain (12.5 mg/mL) solution containing ethanol (0–40% vol.) with an electron beam (0–10 kGy) [8] or γ-irradiation (0–10 kGy) [9]. The authors suggest that irradiation of papain solutions in the presence of EtOH leads to the aggregation of the enzyme and the formation of dityrosine bridges. Dityrosine detection was performed based on measurements of papain fluorescence spectra using λ_exc_ = 325 nm. Schüssler and Davis postulate that the reaction of the hydrated electron with albumin proceeds faster when the HSA solution is pre-irradiated in the presence of ethanol [11,12]. The addition of EtOH to the albumin solution causes partial removal of water molecules from the solvation layer close to the protein surface, which leads to the formation of polypeptide bonds between adjacent albumin molecules. As a result of this process, aggregates form spontaneously before irradiation of the protein solution [7,10,13]. The generation of HSA aggregates in this case consists of two stages: the aggregation of protein molecules caused by the addition of ethanol, followed by radiation cross-linking of HSA molecules, induced by gamma radiation or electron beam. Irradiation of an aqueous solution of ethanol causes the formation of 1-hydroxyethyl radicals (in the publication, we also use CH_3_C^•^HOH interchangeably instead of the 1-hydroxyethyl radical), which, like the hydrated electron, have reducing properties:

HO^•^ + CH_3_CH_2_OH → CH_3_C^•^HOH + H_2_O, (1)

H^•^ + CH_3_CH_2_OH → CH_3_C^•^HOH + H_2_, (2)

The 1-hydroxyethyl radical (uncharged ethanol radical) reacts with organic compounds via three pathways [14]:

1. By electron transfer:

CH_3_C^•^HOH + albumin → albumin^•–^ + CH_3_CHOH + H^+^, (3)

2. By H-abstraction:

CH_3_C^•^HOH + albumin → albumin^•^ + CH_3_CH_2_OH, (4)

3. By addition:

CH_3_C^•^HOH + albumin → albumin^•^(CH_3_)CHOH, (5)

Achilli et al. concluded that the cross-linking process of albumins in the presence of ethanol by the radiation method is a complex process. They irradiated a N_2_-saturated BSA (30 mg/mL) solution containing ethanol (30% vol.) with gamma radiation. In such reducing conditions (in the presence of hydrated electron and ethanol), it is possible to generate carbon-centered radicals. CH_3_C^•^HOH radicals in the absence of oxygen decay mainly via recombination reactions, forming a variety of intramolecular C–S–S–C, C–S–C, and C–C cross-linked bridges within the protein. Moreover, the authors did not observe a peak around 420 nm (λ_exc_ = 325 nm) in the emission spectra of the BSA/ethanol solution after irradiation, which is typical for albumin aggregates under oxidative stress [13]. At the same time, no deamination of BSA amino acid residues was observed. This excludes modifications within the polypeptide chain (the deamination reaction proceeds in two steps: attachment of hydrated electron to the carbonyl group of the peptide bond, followed by elimination of ammonia from the N-terminus of the chain). Considering the important role of disulfide bridges in stabilizing protein nanoparticles, it was suggested that albumin aggregates are formed by recombination of two thiyl radicals or by recombination of the CyS–SCy^•–^ radical with a carbon-centered radical [13].

Considering that the structure of proteins (HSA or BSA) consists of, among others, disulfide-containing residues, several mechanisms of the reaction of reductive species ($e_{aq}^{-},H^{•})\;$with albumins have been proposed. The reaction of albumins with reducing radicals leads to the formation of disulfide anion radicals. Protein aggregation can occur by intermolecular thiol–disulfide exchange. This mechanism involves a nucleophilic attack by a thiolate ion on a sulfur atom in a disulfide of another protein molecule [15]. As a result, a new intermolecular disulfide bond is formed, while maintaining a free thiolate anion that can react with other disulfide bridges. The reaction of the $e_{aq}^{-}$ or $H^{•}\;$leads to the modification of one of the two Cys in the cystine moiety to Ala (alanine residue). This results in the formation of a perthiyl radical [16]. In addition, Cys200, Cys392, and Cys514 are formed as free thiols of CysSH residues. Other authors suggest that CyS–SCy^•–^ radicals, generated in the reaction of CyS–SCy with hydrated electron, recombine in anaerobic conditions, forming intramolecular or intermolecular bonds of the C–S–S–C type, C–S–C (recombination of a thiyl radical with a radical centered on α carbon atom) or C–C (recombination of radicals on the α-carbon atom) [12].

Schüssler et al. postulated that 1-hydroxyethyl radicals in N_2_O-saturated solution of BSA attach covalently to bovine albumin molecules [17]. BSA solutions (1 mg/mL) were irradiated in the presence of 0.1 M ethanol in a N_2_O atmosphere so that the only reacting radicals were 1-hydroxyethyl radicals. The amount of bound ethanol was measured after separation by gel filtration. Moreover, in N_2_-saturated samples of albumin, more (albumin–C(CH_3_)HOH)^•^ radicals were observed compared to the N_2_O-saturated solution [14]. This is probably related to the reaction of the hydrated electron with the protein. The experiment we conducted showed that the hydrogen atom practically does not cause the reduction in the –S–S– bridge inside albumin. The $e_{aq}^{-}$ leads to the formation of BSA radicals, which then react with 1-hydroxyethyl radicals. The authors assumed that in the pulse radiolysis measurements of a 15 μM BSA buffer solution, only 1–2% of all CH_3_C^•^HOH radicals transfer their electrons to the disulfide bonds [12,14].

In general, CH_3_C^•^HOH radicals are not very reactive, even when compared to the reactivity of *tert*-butyl alcohol radicals. For example, the reaction of CH_3_C^•^HOH with cystamine by transferring a hydrogen atom has a rate constant only eight times higher than the analogous reaction constant with the *t*-BuOH radical (1.4 × 10^8^ dm^3^ mol^–1^ s^–1^ versus 1.8 × 10^7^ dm^3^ mol^–1^ s^–1^) [18]. On the other hand, the literature data indicate that irradiation of oxygen-saturated samples of albumin does not lead to a covalent adduct of albumin(CH_3_)CHOH [12].

The aggregation process of albumin induced by the addition of EtOH (desolvation effect) is reversible (dilution of the albumin solution leads to a disaggregation of BSA) [10]. The addition of alcohol leads to desolvation, i.e., removal of water molecules from the nearest solvation layer right next to the protein surface, which results in the formation of polypeptide bonds between albumin molecules. The literature also states that alcohol can bind to the polar groups of albumin, displacing previously bound water [19]. Direct interaction of ethanol with hydrophobic binding sites on fatty acid-free bovine serum albumin (BSA) has also been reported by Avdulov et al. [20]. Based on the fluorescence (using the fluorescent probe 1-anilinonaphthalene-8-sulfonic acid or *cis*-parinaric acid) and NMR measurements, the authors proposed that ethanol directly interacts with hydrophobic binding sites on fatty acid-free bovine serum albumin (BSA). Interestingly, the NMR results suggest that the methyl group of ethanol interacts with the hydrophobic pocket in the protein structure. Sanaeifar et al. investigated gel formation by the addition of different ethanol concentrations to BSA solutions at 37 °C [21]. The addition of high volumes of ethanol enables the formation of hydrogels at 37 °C and neutral pH that are mechanically as robust as those formed by increasing the temperature to 65 °C and lowering the pH to 3.5.

A review of the literature allows us to conclude that albumin nanostructures obtained using radiation methods (diameter 20–70 nm) [8,9] are smaller than analogous ANs obtained using chemical methods (generally above 100 nm) [22]. Radiation-induced synthesis of BSA nanoparticles in the presence of EtOH leads to the formation of nanostructures in the size range 20–70 nm, depending on the alcohol concentration and radiation dose. In another experiment, samples containing different protein concentrations (5–40 mg/mL BSA) and a constant amount of alcohol (40% ethanol vol.) were irradiated. The diameters of the obtained BSA aggregates ranged from 20 to 40 nm, depending on the initial protein concentration [7]. In addition to HSA and BSA, papain nanostructures were also obtained by the radiation method [9]. Papain solutions containing ethanol (0–35% vol.) were exposed to γ-irradiation at a dose of 0–10 kGy. In contrast, the size of BSA nanostructures obtained by a desolvation method using glutaraldehyde as a cross-linker was approximately 86 nm and 92 using glucose [23]. The generated papain nanoparticles under optimized conditions reached sizes ranging from 5 to 13 nm, depending on the ethanol concentration and irradiation dose. In general, from a medical point of view, nanoparticles with a narrow size distribution between 100 nm and 200 nm are optimal for drug delivery [22].

The main objective of this work is to answer the question of whether the addition of ethanol to albumin solutions in the radiation synthesis of protein nanoaggregates brings measurable benefits in terms of process efficiency. Previous efforts in this field have shown the usefulness of ethanol in the synthesis of papain or BSA nanostructures. The literature lacks comparative pulse radiolysis and stationary optical measurements of radical reactions with albumin in aqueous and alcoholic solutions. We are filling this gap.

## 2. Results and Discussion

### 2.1. The Effect of Ethanol on Albumin Structure Before Irradiation

#### 2.1.1. Optical Measurements

The influence of the presence of ethanol on the structure of albumin in aqueous solutions has been studied for years. From these studies, it is known that the percentage of ethanol in water significantly affects many structural parameters of albumins in a non-monotonic manner. It is reported in the literature that as the concentration of ethanol in the HSA solution increases, various changes occur in the albumin structure [24]. As the concentration of EtOH increases (2–30% vol.), the HSA molecule unfolds. Above an ethanol concentration of 30% (vol.), protein aggregation occurs, resulting in the formation of a β-sheet structure. The literature data from absorption and circular dichroism measurements show changes in tertiary and secondary structures of HSA at ethanol concentrations below 30% (vol.) [24,25]. For HSA solutions with ethanol concentrations above 30% (vol.), a change in the α-helix structure to β-sheet and a disordered secondary structure is observed. In addition, protein aggregation occurs. An interesting phenomenon is that for ethanol concentrations higher than 55% (vol.), protein aggregates deaggregate.

**Figure 1:**
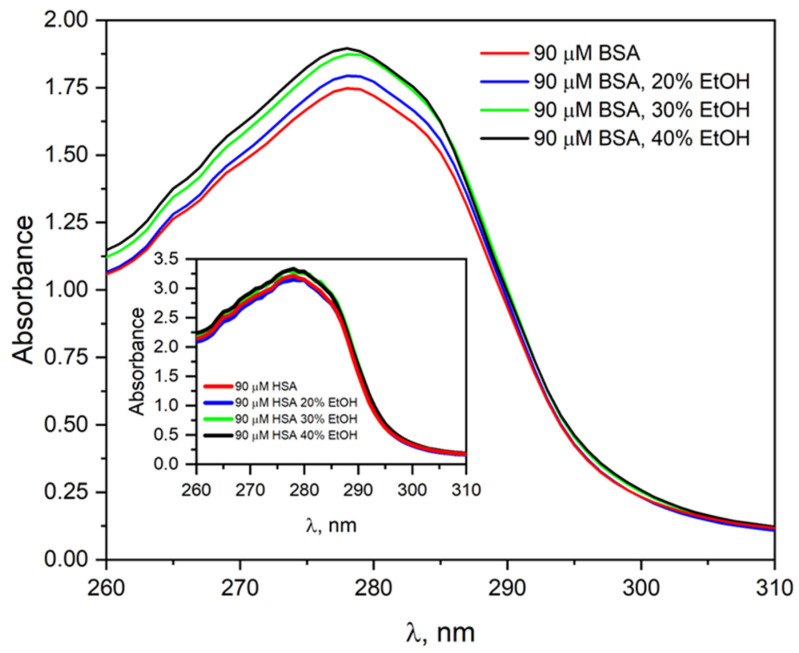
Absorption spectra of the neat BSA solution (90 µM) and containing 20, 30, and 40% EtOH (vol.). Inset. Absorption spectra of the HSA solution (90 µM) before and after adding different amounts of ethanol: 20, 30, or 40% (vol.).

The effect of ethanol on the albumin’s structure was investigated by using absorbance and fluorescence spectroscopy. Measurements were made for a neat BSA (Figure 1) or HSA solution (Inset in Figure 1) before and after adding ethanol. Figure 1 compares absorbance spectra recorded for BSA solution in the absence and presence of different amounts of ethanol: 20, 30, and 40% (vol.). The absorption spectrum of BSA shows a band with a maximum at 278 nm. The addition of EtOH to the albumin solution leads to a slight increase in absorption at the maximum of this band. In the HSA solution containing the same amounts of ethanol, similar changes in the absorption spectrum were observed (Inset in Figure 1). The absorbance value at the maximum of the HSA band increases by 4% compared to the neat albumin solution without added ethanol.

**Figure 2:**
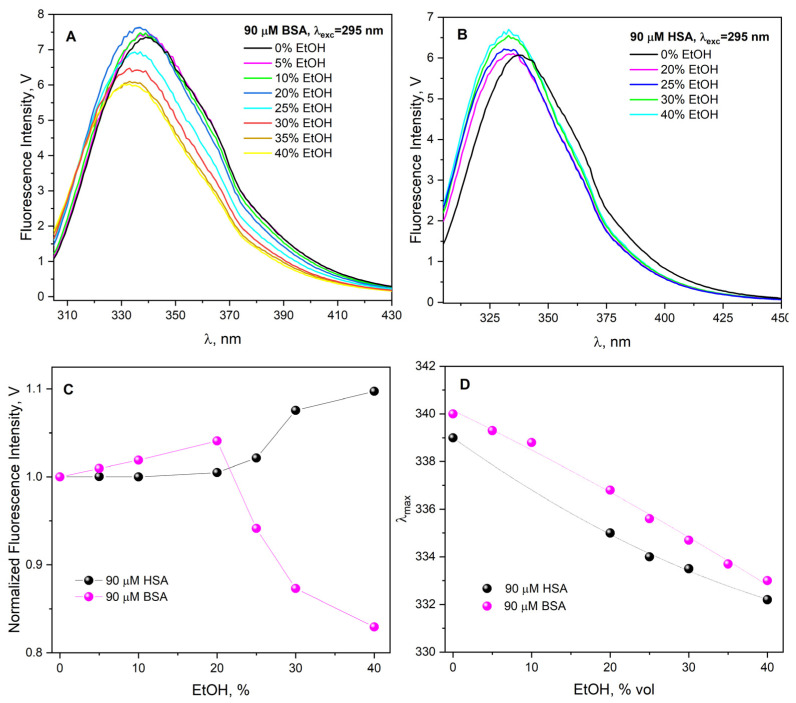
(**A**) Emission spectra of an aqueous solution of BSA (90 µM) recorded before and after adding EtOH (0–40% vol.). (**B**) Emission spectra of an aqueous solution of HSA (90 µM) recorded before and after adding EtOH (0–40% vol.). (**C**) Normalized maximum of fluorescence intensity of Trp in 90 µM HSA solution (black dots) and in 90 µM BSA solution (magenta dots) as a function of EtOH concentration. In both cases, the excitation wavelength was 295 nm. (**D**) The dependence of λ_max_ (maximum of the fluorescence of tryptophanyl residues) for HSA and BSA as a function of EtOH concentration.

The effect of ethanol on the fluorescent properties of BSA (see Figure 2A) and HSA (Figure 2B) was also observed. Albumin fluorescence intensity varies differently as a function of ethanol concentration (Figure 2C). The addition of EtOH to the albumin’s aqueous solution caused a hypsochromic shift of the Trp fluorescence band (for BSA 340 nm → 333 nm, for HSA 337 nm → 333 nm; λ_exc_ = 295 nm) (Figure 2D).

For both proteins, no significant changes in fluorescence intensity take place up to ethanol concentration about 20–25% vol (Figure 2C). In the case of EtOH concentrations above 30% by volume of the studied solutions, the fluorescence of Trp residues in albumins shows a different behavior: the fluorescence intensity of BSA decreases, and that of Trp214 (HSA) increases (Figure 2C). The increase in Trp214 fluorescence intensity means that the addition of ethanol to the albumin solution leads to a change in the microenvironment within site 1 where this residue is located. These changes may be due to a change in the polarity of the ethanol-containing solution within albumin (compared to an aqueous solution of albumin) and/or to slight changes in the structure of site 1 induced by the presence of EtOH. Changes in the fluorescence spectra of ethanol–water solutions of BSA are more complex. In contrast to aqueous solutions containing more than 20% EtOH and HSA, for which an increase in Trp214 efficiency is observed, BSA solutions are characterized by a decrease in the fluorescence intensity of the solutions. Our measurements are in agreement with the literature [26,27]. This is most likely related to the existence of two different BSA domains, in which two tryptophan groups (Trp134, Trp213) are located.

No significant changes in the shape of albumin fluorescence spectra and their intensity (excitation at 295 nm) were observed between 0% and about 25% ethanol, indicating a slight impact of such low ethanol concentrations on the secondary structure of HSA or BSA. This is consistent with the conclusions regarding the influence of the presence of ethanol on the structure of albumin, also examined using other methods, including DLS (Dynamic Light Scattering) or CD (far UV circular dichroism) [24,28]. The circular dichroism data [24] regarding the effect of ethanol on the structure of HSA in aqueous solutions are in good agreement with the results of our spectroscopic measurements. This applies in particular to the threshold EtOH content at which the formation of HSA aggregates is observed. The work [24] showed that in the case of solutions with EtOH content below 30% by volume, the changes in the HSA structure are moderate. A clear, abrupt change in the α-helix and β-sheet content was observed in the range from 30 to 40% of EtOH. The alpha helix content decreased from 66.6% to 41.8%, and the beta sheet content increased from 3.8% to 11.3% in the composition of the HSA secondary structure. This can be associated with enhanced intermolecular interactions between β-strands of HSA, and protein aggregation starts to occur.

**Figure 3:**
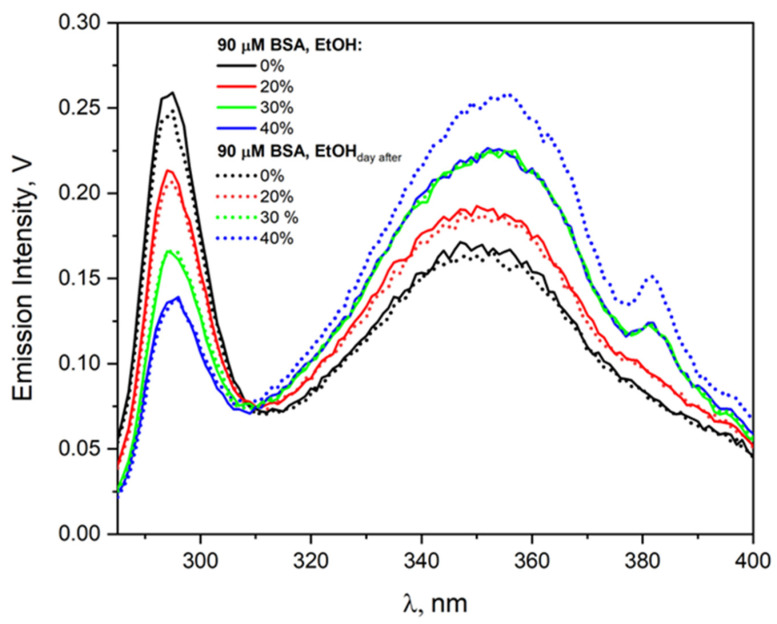
Emission excitation spectra of the BSA solutions (90 μM) containing different amounts of ethanol: 0, 20, 30, or 40% (vol). The emission detection wavelength was 430 nm.

Figure 3 shows the excitation spectra recorded for the emission band at 430 nm for a BSA solution (90 µM) containing different amounts of ethanol: 0, 20, 30, or 40% (vol.). In the excitation spectrum of neat BSA solution, two bands with a maximum at 295 nm and about 350 nm were observed. The band centered at 295 nm can be attributed to the Trp residues, while the band with a maximum at 350 nm is characteristic of spontaneous BSA aggregates. In our previous work [29] on the formation of spontaneous aggregates of BSA and HSA, we clearly explained that the band with a maximum at 295 nm is derived from tryptophanyl residues, and the aggregates are responsible for the broad emission spectrum centered at about 350 nm. Hydrogen bond networks with a β-sheet cross-section structure and/or π-π stacked interactions cause the formation of aggregates as a source of intrinsic blue autofluorescence (maximum emission spectrum above 400 nm not observed for very low protein concentrations) in aqueous HSA or BSA solutions. After the addition of alcohol to the albumin solution, the same number of bands in the excitation spectra was observed for an ethanol concentration of 20–30% vol.; however, the intensity of the bands centered at 350 nm increases significantly compared to the neat BSA solution (solid lines).

The intensity of the emission band of Trp residues decreases, which indicates the deactivation of the excited state of the Trp molecule due to the transfer of energy from ^*^Trp to BSA aggregates formed by the addition of ethanol [29]. In the case of the BSA solution containing 40% ethanol, the appearance of a third band with a maximum at 382 nm in the excitation spectrum was observed. The origin of this band (382 nm) requires explanation using the DLS technique. After 24 h, the excitation spectra of these solutions were recorded. Only in the case of the BSA solution containing 40% ethanol was a significant increase in emissions intensity observed. Analogous experimental results were obtained for HSA solution containing ethanol in the concentration range of 0–40% vol. The conclusions from both experiments (for BSA and HSA solutions) are the same and indicate that only solutions with ethanol concentration above 30% by volume become unstable. Our measurements are in good agreement with the literature. Zhdanova et al. concluded that the aggregation process of HSA in ethanol solution starts at [EtOH] = 40% (vol.) [8,26,27]. Based on measurements of Trp and Tyr fluorescence in HSA and BSA, the authors suggested that protein conformational changes at low ethanol concentrations occur in domain III of albumin, which does not contain tryptophan residues.

#### 2.1.2. Time-Resolved Fluorescence Measurements

**Figure 4:**
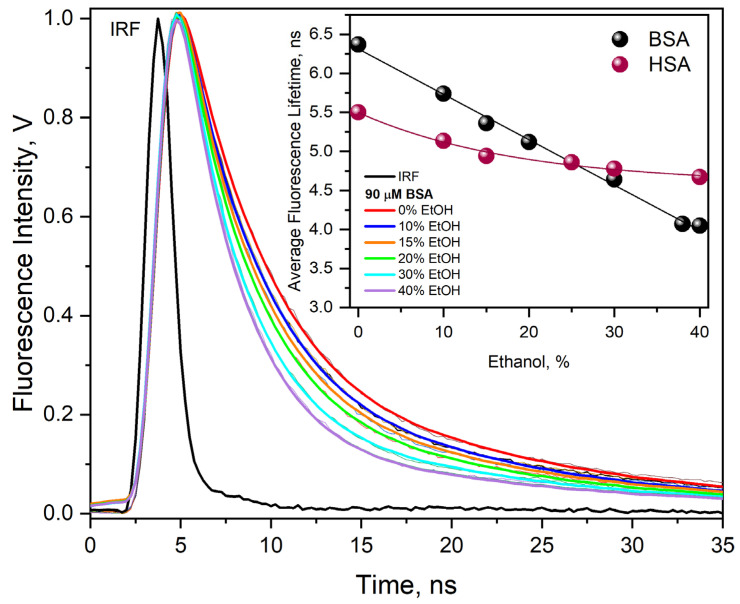
Decays of fluorescence of the neat BSA solution (90 µM), a solution of BSA (90 μM) containing various amounts of EtOH 0–40% (vol.), and biexponential best fits to experimental curves. IRF (instrument response function) for a 295 nm LED excitation source. Inset. The dependence of the average value of fluorescence lifetime of BSA or HSA as a function of EtOH concentration (0–40% vol.).

The time-resolved fluorescence measurements for BSA solution before and after the addition of ethanol revealed that there is a decrease in the lifetime of Trp with increasing ethanol content (0–40% vol.) (Figure 4). The experiment confirmed that the addition of ethanol leads to such a modification of the BSA structure that Trp residues are exposed to the water phase. In the case of BSA, a linear decrease in albumin fluorescence lifetime was also observed with increasing ethanol concentration, but this dependence is not linear for the HSA solution. This is probably due to the difference in the number of Trp residues in the structure of both proteins (HSA has one Trp residue, BSA has two). The tryptophan fluorescence, as well as tryptophan residue fluorescence in proteins, is sensitive to the microenvironment in general, and particularly to polarity. The shift of the fluorescence maximum of tryptophan residues in albumins towards shorter wavelengths (inset D to Figure 2) indicates a lower solution polarity in their microenvironment. The differences in protein fluorescence behavior due to the presence of ethanol can be attributed to the fact that Trp-134 occurs exclusively in BSA, assuming that Trp213 in BSA behaves the same as Trp214 in HSA. As can be seen in the inset of Figure 4, in the case of Trp214 (HSA), with the increase in ethanol concentration in the solution, the fluorescence lifetime gradually decreases and reaches a plateau at an average time of 4.7 ns.

A more pronounced decrease in the BSA fluorescence lifetime (average time of about 4 ns for the water–EtOH solution (40% vol.)) can therefore be attributed to Trp 134. Trp-134 is more exposed to solvent because it is located on the BSA surface in the hydrophilic IB subdomain, but determining whether this is the main reason for the differences in the temporal deactivation of the excited states of HSA and BSA requires further study. The addition of ethanol causes an increase in the fluorescence intensity of Trp214 while shortening the emission duration. This means that the presence of ethanol affects the fluorescence lifetime of the tryptophan residue, as well as the structure of the HSA domain in which Trp214 is located.

#### 2.1.3. NIR and RLS Measurements

Recently, we investigated the influence of albumin concentration on the three-dimensional structure of water using near-infrared spectroscopy [29]. No changes in the water structure were observed in the presence of HSA at the physiological concentration (600 μM). In this paper, we also recorded NIR spectra (see Figure S1 in Supplementary Materials) in the range from 1350 to 2140 nm for several solutions containing water and ethanol with different EtOH contents (0–40 vol%). We compared the NIR spectra of EtOH/water solutions with the spectra of solutions additionally containing HSA. The addition of HSA (Figure S1 in Supplementary Materials shows data for 300 µM HSA and 30% EtOH) does not affect the NIR spectra. However, for EtOH concentrations of 40% and more, the NIR spectra change. In this range, the baseline of the spectra increases significantly, and the increase in absorbance is associated with the formation of HSA aggregates and turbidity of the solutions.

Resonance light scattering (RLS) is a sensitive and useful technique for detecting protein aggregates [30]. RLS spectra are recorded using a classical spectrofluorometer by simultaneously scanning the excitation and emission monochromators of a typical spectrofluorometer at Δλ = 0. The RLS spectra of BSA solutions in neat water and a solution containing 20, 30, or 40% EtOH are shown in Figure S2 in the Supplementary Materials. The spectra were recorded twice with an interval of 24 h. The spectrum changed only for the binary water/ethanol solution with a concentration of 60/40% by volume. A significant increase in the RLS signal intensity for the 40% EtOH solution indicates the spontaneous formation of aggregates composed of BSA molecules.

### 2.2. Reaction of 1-Hydroxyethyl Radicals with Albumins

Radiolysis of Albumin Solution

**Figure 5:**
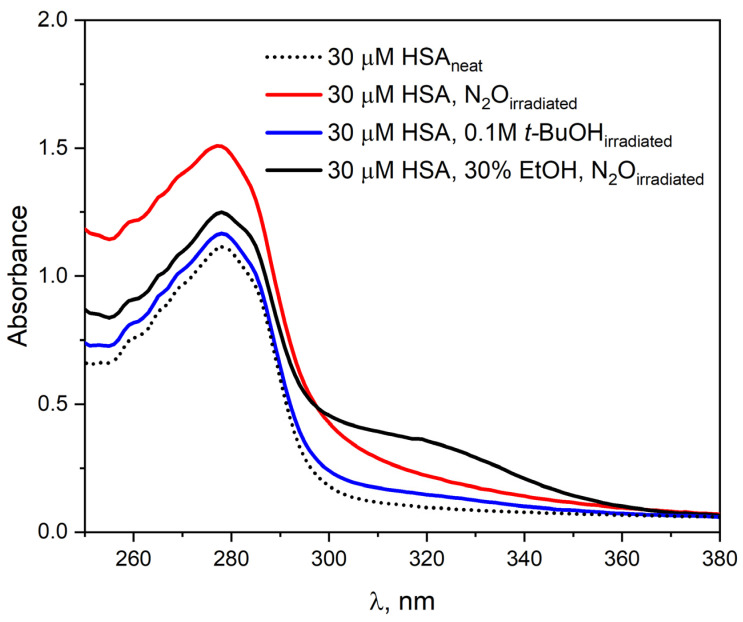
Absorption spectra of the HSA solution (30 µM) before irradiation (dotted line); saturated N_2_O after irradiation (red line); containing *t*-BuOH (0.1 M) after irradiation (blue line); containing 30% EtOH and saturated N_2_O after irradiation (black line). All solutions were irradiated with a dose of 4200 Gy.

To investigate the effect of ethanol on the absorption properties of HSA, the pulse radiolysis system was used to irradiate a 30 µM HSA solution (4200 Gy) in the presence of EtOH (30% vol.). Figure 5 shows the HSA absorbance spectra before and after irradiation.

Before irradiation, the absorption spectrum of HSA is characterized by a single band with a maximum at 278 nm. The addition of EtOH to the HSA solution leads only to a slight increase in absorbance at the maximum of this band. After irradiation of N_2_O-saturated albumin solution with ethanol, an increase in the intensity of the HSA band and the appearance of a new absorbance in the spectral range of 295–375 nm were observed. The new absorbance in the albumin spectrum after irradiation of an HSA solution containing ethanol indicates the formation of new species in the sample that specifically absorb light. The apparent increase in absorbance at the maximum of the HSA band after irradiation of the solution is related to light scattering by the sample. The absorption spectra of albumin after irradiation of a solution with added ethanol differ from those recorded after the reaction of HSA with hydrated electron. The differences in the emission spectra may result from the fact that the addition of ethanol already in the initial solution modifies the structure of HSA, and also that the addition of CH_3_CH^•^OH radicals to HSA molecules was possible. Figure 5 also shows the HSA spectrum related to steady-state irradiation of N_2_O-saturated albumin solution. The experimental results indicate that the reaction of 1-hydroxyethyl radicals does not lead to the formation of a protein oxidation product.

Figure S3 shows the emission spectra recorded before and after pulse radiolysis of an HSA solution (300 µM) containing *t*-BuOH (0.1 M) or a solution containing HSA (300 µM) and 20% EtOH (vol.) saturated with N_2_O or vacuum-deaerated. In the case of vacuum-deaerated EtOH solution, the emission spectrum of HSA is identical to that of a solution with *t*-BuOH. This is obvious because in such deaeration conditions, mainly the hydrated electron reacts with albumin. The intensity of the band with a maximum at around 420 nm in the emission spectrum of HSA after irradiation of a solution containing EtOH and saturated with N_2_O is definitely lower compared to the solution containing *t*-BuOH. Under such conditions, the reducing species is the 1-hydroxyethyl radical, and the reactivity of those radicals is lower than that of hydrated electrons. However, analyzing the HSA emission spectra in Figure S3, it can be concluded that the albumin reduction product after reaction with a hydrated electron or CH_3_C^•^HOH radical is the same in both cases. To confirm the reductive properties of the 1-hydroxyethyl radical, a pulse radiolysis experiment of HSA solution under oxidative conditions (the oxidizing species was the ^•^OH radical) was performed. The emission spectrum of albumin recorded after reaction with the hydroxyl radical is red-shifted (5 nm) and its intensity is almost two times higher (red line in Figure S3) compared to the emission spectrum of the HSA solution with *t*-BuOH. This excludes the oxidation reaction of proteins with CH_3_C^•^HOH radicals.

**Figure 6:**
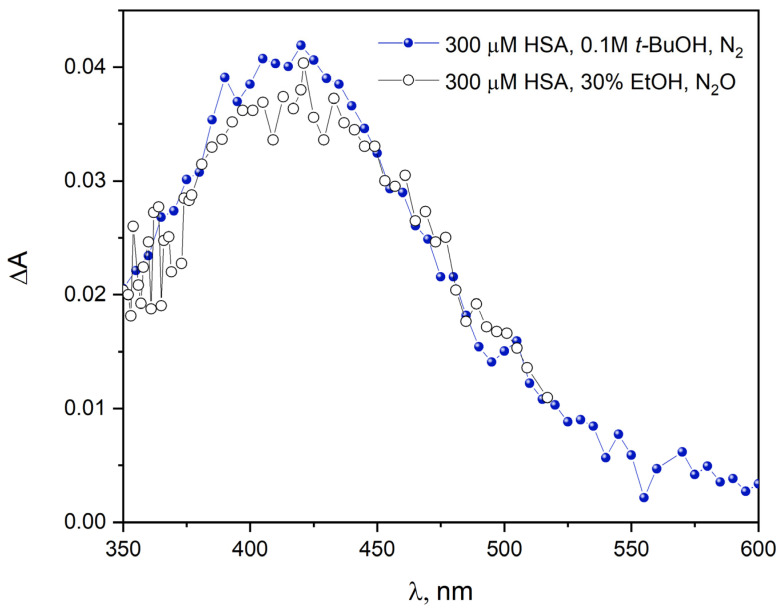
Transient absorption spectrum: N_2_-saturated buffer solution containing 300 μM HSA and 0.1 M *t*-BuOH, obtained for irradiation dose 33 Gy; normalized spectrum of N_2_O-saturated aqueous solution containing 300 μM HSA and 30% EtOH (vol.), obtained for irradiation dose 65 Gy.

We studied the reactions of uncharged ethanol radical with human serum albumin (HSA) in pulse radiolysis (Figure 6). Figure 6 shows normalized transient absorption spectra of primary products of HSA reduction by 1-hydroxyethyl radical (N_2_O-saturated 300 µM HSA solution containing 30% EtOH). These spectra are compared with the spectra obtained after the reduction in HSA by hydrated electron (N_2_-saturated 300 µM HSA solution containing 0.1 M *t*-BuOH). In the pulse radiolysis measurements of the HSA solution containing ethanol, we used 300 µM HSA because at a concentration of 30 µM HSA, no reaction of CH_3_C^•^HOH radicals with albumin was observed. Trace amounts of reduced HSA used at a concentration of 30 µM were detected only in the case of very high doses (over 800 Gy) generated by 4 µs accelerator electron pulses. The analysis of the shape of transient absorption spectra of N_2_O-saturated HSA solution containing 30% EtOH gave preliminary evidence for the mechanism of the reactions of 1-hydroxyethyl radicals with human serum albumin. The new transient absorption peak with a maximum at 420 nm is formed after pulse irradiation. This spectrum can be attributed to the reduction product of the disulfide bond (CyS–SCy^•–^) within albumin. Similar changes have been observed in the case of reduction in the HSA by hydrated electron [5,31]. The intensity of the transient absorption spectrum of HSA in the case of 1-hydroxyethyl radicals is 3.2 times lower compared to the spectrum of the protein after the reaction with the hydrated electron (in this work, we show the normalized spectra). This is most likely due to the lower driving force of the CH_3_C^•^HOH reaction with –S–S– bridges within the HSA compared to the analogous reaction of $e_{aq}^{-}$ with HSA.

The hydrated electron is an extremely effective reducing agent with a reduction potential of –2.9 V. The redox potential of the 1-hydroxyethyl radical is –1.1 V. Another reason for the difference in absorbance at 420 nm generated by the hydrated electron or the CH_3_C^•^HOH radical could be the fact that some of the 1-hydroxyethyl radicals formed in solution can bind to HSA. A similar mechanism of the reaction of 1-hydroxyethyl radicals with BSA was proposed by Schüssler in 1981 [17]. It was concluded that initial reactions of the organic radicals result in a conformational change, which makes hidden groups accessible. Comparison of normalized absorption spectra (Figure 6) recorded during pulse radiolysis of aqueous HSA solutions saturated with N_2_ (300 µM) containing *t*-BuOH (0.1 M, the reactive species is the hydrated electron) or 30% EtOH vol. (under such conditions, there are two reactive species in the sample: the hydrated electron and the 1-hydroxyethyl radical) indicates that in both cases the reduction in cystine residues of albumin occurs. We did not observe the formation of CH_3_C^•^HOH radical adduct to the HSA polypeptide chains.

The decay of CyS–SCy^•–^ in the solution of HSA in the absence and presence of ethanol was also monitored by the pulse radiolysis technique. The absorbance was recorded at 420 nm. The decay of the disulfide radical anion within the HSA structure in both cases is a slow process and takes place on a time scale of seconds (Figure S4). The kinetics of this band are described by triexponential decay, which is due to initial attachment at different disulfide linkages that have various intrinsic lifetimes. The trace at 420 nm evolves with the same kinetics in both cases. In our opinion, the disappearance of the reduced –S–S– bridges shown in Figure S4 is related to the formation of hydrogels (under certain conditions, at high albumin concentration, appropriate frequency of ionizing radiation deposition, appropriate solution temperature). To understand the mechanisms of HSA or BSA aggregate formation, including hydrogels, it is necessary to use the scattered laser light detection method combined with the pulse radiolysis technique. We intend to carry out such measurements soon, using our own appropriate equipment. We have shown that under conditions of reductive stress (selectively generated by ionizing radiation, hydrated electrons, or CO_2_^•–^ anion radicals) stable protein aggregates can be formed in solutions containing HSA and 0.1 M *t*-BuOH. Albumin solution concentrations must be high, close to 300 μM or higher. We reported the possibility of preparing hydrogels at room temperature and at temperatures elevated up to 60–70° C. The key issue for the effective preparation of hydrogels is the method of delivering the electron beam to the neat albumin solutions. In this work, the preparation of albumin aggregates using an electron accelerator for neat solutions containing ethanol was compared with solutions containing 0.1 M *t*-BuOH. The general conclusion is that using ethanol concentrations below 40% vol. in albumin stock solutions does not provide any advantage over preparing hydrogels by irradiation using 0.1 M *t*-BuOH. Work on the optimization of the hydrogel preparation procedure (pH, solution temperature, albumin concentration, electron beam repetition rate, absorbed dose, etc.) is time-consuming and requires continuation. Certainly, the radiation method of generating albumin aggregates leads to interesting biocompatible materials for medical applications. In some experiments, when the solutions were deaerated by vacuum, clear, transparent gels were obtained. A picture of cuvettes containing albumin hydrogel produced by electron beam is shown in the graphical abstract. Photographs of human serum albumin gels obtained after electron beam irradiation of an aqueous HSA solution containing 0.1 M *t*-BuOH (left photo) or an irradiated HSA solution containing a large excess of ethanol (right photo). Air was removed from the solutions by vacuum, and the HSA concentration was 300 µM. In our opinion, only for these conditions (excessive proportion of ethanol in relation to water and HSA or BSA concentration above 300 µM), the use of ionizing radiation gives hope for the commercial production of AN. Certainly, the radiation method, which is effective in protein desolvation, is more advantageous than the method in which chemical reducers are used.

The lifetime of hydrated electrons in irradiated aqueous buffer solution and solution containing EtOH is the same and is equal to 3.0 μs. It is well known that the hydrated electron practically does not react with alcohol molecules, including EtOH. Since the beginning of the use of the pulse radiolysis method, the reactions of hydrated electrons with scavengers of various efficiencies have been studied depending on the composition of the solution, which was, among others, a mixture of water and ethanol [32,33]. Differences in kinetic behavior reactions of solvated electrons with scavengers in water and aqueous solutions with different ethanol content are caused by differences in the values of reagent diffusion coefficients [34], solvation energies [35], and dielectric properties of the binary medium [33].

**Figure 7:**
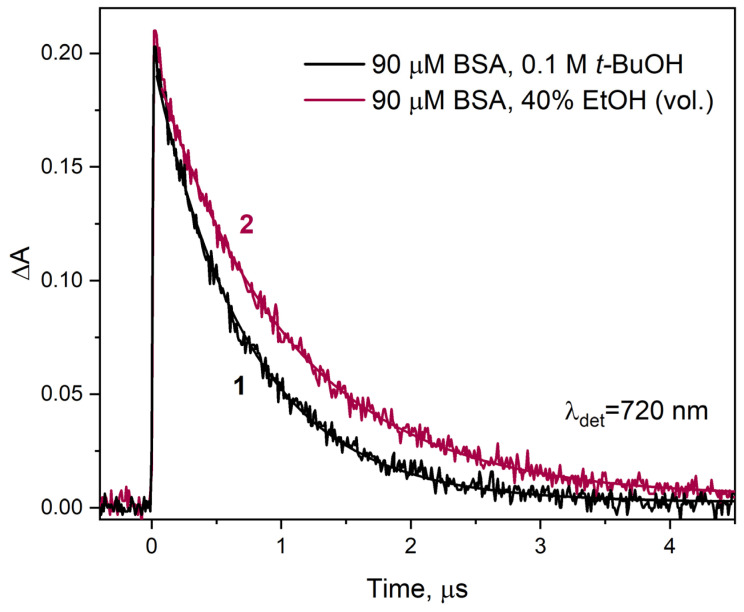
Time profiles of the absorbance recorded at 720 nm after 17 ns pulse irradiation with a dose of 33.9 Gy of the vacuum deaerated aqueous solutions: containing BSA (90 µM) and *t*-BuOH (0.1 M) (curve 1) or BSA (90 µM) and EtOH (40% vol. curve 2). The smooth lines represent monoexponential fits to experimental curves (noisy ones).

The presence of ethanol in the irradiated aqueous solution also affects the structure of the electron solvation shell. In binary solutions, the electron has different properties than those generated during the radiolysis of water and should be called solvated, not hydrated. When comparatively analyzing the decay of electrons in the reaction with HSA in water and in an aqueous solution containing a specific, not too large, amount of ethanol, all the factors mentioned above should be taken into account. The highest proportion of ethanol in our pulse radiolysis measurements was for a solution containing forty percent alcohol by volume. Therefore, this solution contains 17.1 mol (corresponding to 40% vol.) EtOH, and the alcohol concentration is 6.85 mol × dm^−3^. To follow the disappearance of solvated electrons upon reaction with BSA in binary solution (40% EtOH vol.), we recorded the transient electron absorbance at 720 nm as a function of time (Figure 7). The decay time of the solvated electron absorption band in this case was 1.03 µs (curve 2). In a solution containing BSA without ethanol, the hydrated electron persists for a shorter time; the decay time is 720 ns (curve 1).

Based on the literature data mentioned above, it can be assumed that the difference in electron reactivity in the reaction with BSA in the presence and absence of EtOH is related to the change in the properties of the binary solution in relation to neat water. We believe this is not the only reason. The aggregation of albumin due to desolvation has a greater impact on the efficiency of the one-electron reduction in BSA. This is manifested by a decrease in the reaction rate constant $e_{sol}^{-}$ with BSA with time and aging of the solution. A 40% solution of BSA in ethanol, subjected to pulse radiolysis five hours after preparation, had a solvated electron decay time of over one and a half microseconds (in contrast to 1.03 µs for a freshly prepared sample). We analyzed the decays of the solvated electron absorption band for solutions of 90 µM BSA and containing 5, 10, 20, 30, and 40% EtOH. In the publication, we presented data from pulse radiolysis only for 40% EtOH (Figure 7). Measurements performed at an interval of 24 h gave identical kinetics of the decay of the absorbance of $e_{sol}^{-}$ and those of the scavenger product recorded at 420 nm for all ethanol contents except for the sample containing 40% EtOH (vol.). The decrease in the efficiency of the –S–S– bridge reduction in the protein interior associated with the formation of albumin aggregates was noted by analyzing the intensity of the absorption band with a maximum of 420 nm. Example kinetic data from pulse radiolysis measurements are presented in Figure S5 in the Supplementary Materials. Here, as the solution ages (measurement after 5 h and after a day), the intensity of the –S–S– band decreases, but only for the solution with an alcohol concentration of 6.85 M (40% EtOH vol.). Of course, solution parameters such as viscosity and dielectric constant did not change during this time. Aggregation of HSA or BSA induced by the desolvation process of solutions above 40% EtOH (vol.) reduces the number of isolated, single albumin molecules. This causes a decrease in the concentration of the electron scavenger, which prolongs the lifetime of the solvated electron and reduces the efficiency of protein reduction. We demonstrated the instability of albumin solutions containing more than 40% ethanol using emission measurements (Figure 3 shows increase in the intensity of the emission band of BSA aggregates after one day of solution storage), NIR measurements (see Figure S1 in Supplementary Materials), and RLS measurements (see Figure S2 in Supplementary Materials).

**Figure 8:**
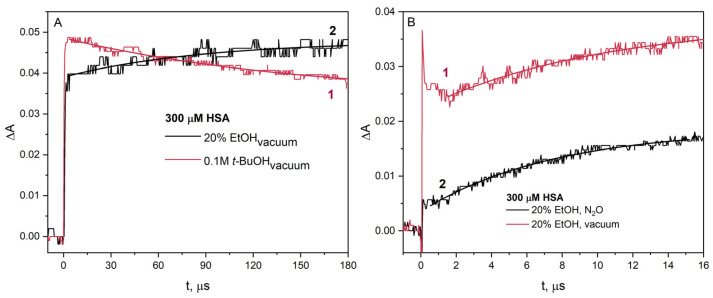
(**A**) Time profiles of the absorbance recorded at 420 nm after irradiation with a dose of 34 Gy of the vacuum-deaerated HSA (300 M) solution containing 20% EtOH or 0.1 M *t*-BuOH. The kinetics of these oscilloscope patterns are described by monoexponential decay (curve 1) and monoexponential buildup (curve 2). (**B**) Time profiles of the absorbance recorded at 420 nm after 17 ns pulse irradiation with a dose of 38 Gy of the vacuum deaerated (curve 1) or N_2_O-saturated aqueous solution containing HSA (300 µM) and EtOH (20% vol.) (curve 2).

For a more detailed analysis of the reaction of the CH_3_C^•^HOH radical with HSA, we performed pulse radiolysis experiments under various conditions. Samples subjected to electron beam irradiation containing *t*-BuOH or EtOH as scavengers of ^•^OH radicals were deaerated under vacuum. Under such conditions, the reactive species in the solution containing *t*-BuOH is the hydrated electron, and in the solution containing EtOH, there are both the hydrated electron and 1-hydroxyethyl radical (Figure 8A). The difference is in the intensity of absorption of the transient recorded (ΔA_0_) immediately after irradiation of the HSA solution containing *t*-BuOH (ΔA_0_ is about 0.05) or EtOH (ΔA_0_ is about 0.04).

The difference in absorbance value is due to protein aggregation in the presence of ethanol before irradiation. The amount of monomeric albumin decreases due to the formation of spontaneous HSA aggregates and causes a lower initial absorbance value in the pulse radiolysis experiment. In the case of pulse radiolysis of HSA solution containing *t*-BuOH, the signal coming (curve 1) from the reduced –S–S– bridge (spectral maximum of 420 nm) disappears in the analyzed time interval. In the radiolysis experiment using EtOH as an ^•^OH radical scavenger, an increase in the signal of the HSA reduction product recorded at 420 nm was observed (curve 2; Figure 8A). The increase in absorbance observed at 420 nm (curve 2) is due to the reduction in –S–S– residues of albumin as a result of the reaction of HSA with radicals CH_3_C^•^HOH. On the long time scale, the decays of the one-electron reduction product of HSA in solutions containing EtOH or *t*-BuOH are very similar (see Figure S4). This experiment confirms that EtOH used at low concentrations behaves as a classical ^•^OH radical scavenger, namely *t*-BuOH.

The kinetic absorption pattern for HSA solution containing ethanol at 420 nm is complex. Recombination of 1-hydroxyethyl radicals also plays a crucial role in the HSA aggregation. 1-hydroxyethyl radicals generated in a water/EtOH solution (70/30% vol.) persist for about 150 µs for a dose of 200 Gy and decay by mutual recombination (see Figure S6 in Supplementary Materials). 1-hydroxyethyl radicals recombine at a rate constant of 2k = 1.2 × 10^9^ M^–1^ s^–1^, which we calculated using second-order kinetics for the decay in Figure S6 in Supplementary Materials and the determined value of the molar absorbance coefficient at a wavelength of 260 nm of 800 dm^3^ mol^–1^ cm^–1^ for the CH_3_C^•^HOH radical. For this reason, the concentration of available –S–S– groups must be rather high, so that the reduction in disulfides becomes measurable. Despite the complex kinetics, we estimated the rate constant of the reaction of the CH_3_C^•^HOH radical with HSA. The rate constant of this reaction is equal 7 × 10^8^ dm^3^ mol^–1^ s^–1^.

The next stage of our research was to study the reaction of the CH_3_C^•^HOH radical with the –S–S– bridges in the HSA structure. For this purpose, we conducted pulse radiolysis experiments of HSA solutions (300 µM) containing EtOH (up to 30% vol.) and saturated with N_2_O or vacuum-deaerated. If an albumin solution containing EtOH was irradiated after N_2_O saturation, only CH_3_C^•^HOH radicals react with the protein molecules. In vacuum-deaerated aqueous solutions, the reducing agents are both $e_{aq}^{-}$ and CH_3_C^•^HOH radical. As a result of N_2_O saturation of albumin solution, the hydrated electrons are converted to ^•^OH radicals and then to CH_3_C^•^HOH radicals. For this reason, irradiation of aqueous solutions with the same dose results in the concentration of the CH_3_C^•^HOH radical being twice as high as the concentration of the hydrated electron. Assuming that all CH_3_C^•^HOH radicals are scavenged by HSA, the concentration of modified –S–S– bridges in the N_2_O-saturated solution should be twice as high as in the vacuum-deaerated solution. However, this was not the case (Figure 8B). This is due to the low value of the CH_3_C^•^HOH radical redox potential and other channels of their decay (reactions numbers 3–5). This may indicate that under such measurement conditions, CH_3_C^•^HOH radicals also attach to the HSA structure. The works of Schüssler [14,17] show that 1-hydroxyethyl radicals undergo an addition reaction to albumin during radiolysis. The access of CH_3_C^•^HOH radicals to disulfide bridges in HSA molecules is difficult, and these radicals may react mainly on the surface of the protein with the primary radicals:

e_aq_^–^ + albumin → albumin^•^, (6)

CH_3_C^•^HOH + albumin^•–^ → albumin(CH_3_)CHOH, (7)

Curve 2 in Figure 8B, showing the increase in absorbance of the reduction product of selected –S–S– bridges in albumin, is clear evidence of electron transfer from the radical CH_3_C^•^HOH to HSA. In the case of pulse radiolysis of a solution of identical composition from which air has been removed, a significant increase in transient absorbance is observed. Obviously, this is due to the participation of hydrated electrons in the reduction of albumin. In the vacuum-deaerated solution, in addition to hydrated electrons, 1-hydroxyethyl radicals are also generated, resulting in an increase in the transient absorbance recorded at 425 nm. This increase in absorbance is caused by the reaction of HSA with the 1-hydroxyethyl, and the growth kinetics corresponds to that of the HSA reaction with only CH_3_C^•^HOH radicals.

**Figure 9:**
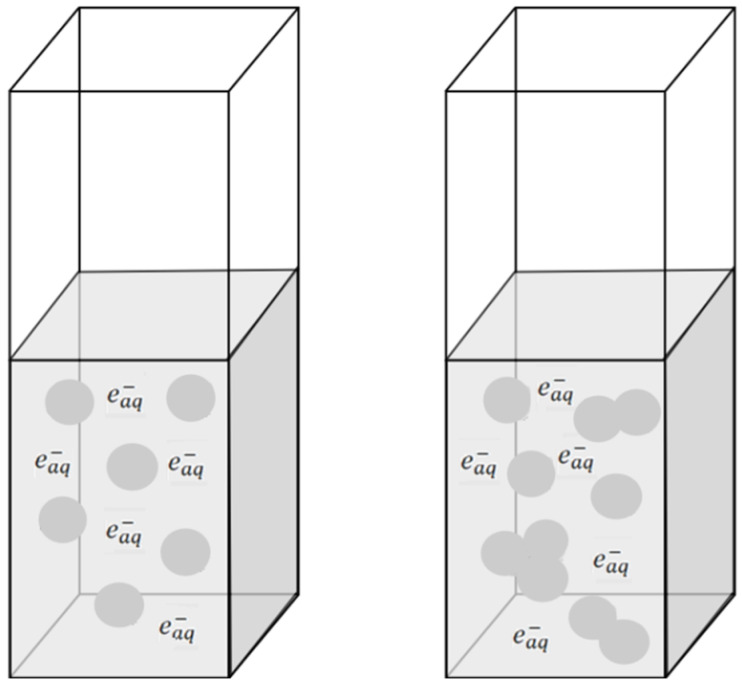
Schematic representation of electron scavenging by albumin: (left cuvette) without ethanol, the effective electron capture volume (circles) by n HSA molecules (distributed uniformly in solution is equal to n·V_aq_ where V_aq_ is the spherical capture volume of isolated HSA); (right cuvette) in the presence of ethanol the effective electron capture volume V_EtOH_ is smaller than n·V_aq,_ which is caused by overlapping of the scavenging spheres of albumin molecules firmly sticked (non-uniform blobs).

Under reducing conditions, the addition of ethanol does not break more disulfide bonds compared to a solution in which –S–S– bonds are reduced only by the hydrated electron or formate radical (regardless of the concentration of HSA) [11]. Our experimental results showed that the reaction efficiency of hydrated electrons with the same amount of HSA decreases in albumin solution containing EtOH compared to the solution containing 0.1 M *t*-BuOH. In the case of HSA solution containing EtOH, the amount of monomeric albumin decreases due to the spontaneous formation of HSA aggregates as a consequence of the addition of alcohol to the solution (Figure 9).

Albumin molecules are surrounded by layers of water. The addition of alcohol to the protein solution changes the hydration of the albumin, particularly affecting the first layer of water molecules surrounding the HSA. The literature reported the favorable interaction of ethanol with hydrophobic residues (leading to protein denaturation) but the unfavorable interaction with charged residues (causing reduction in protein solubility) [28]. The presence of EtOH in the protein solution leads to the formation of polypeptide bonds between albumin molecules [7,10,13].

It is important to note that the generation of HSA aggregates in a solution containing ethanol, therefore, consists of two steps: the ethanol-induced spontaneous aggregation of protein molecules and radiation cross-linking within HSA molecules induced by gamma radiation or electron beam. The HSA aggregates are the result of the recombination of –S–S^•–^ radicals. This leads to the formation of intermolecular –S–S– bridges between HSA molecules, and it is also possible to generate other covalent bridges (including C–C and C–S bonds) [13].

The reaction of reactive species with albumins can influence the structural integrity of proteins and lead to changes in their fluorescence lifetime. In order to more precisely analyze the process of HSA molecule aggregation in the presence of alcohol, time-resolved measurements of the emission decay of the studied solutions were performed (Figure S7). The experiment for non-irradiated HSA solution (20 μM) after the addition of ethanol (30% vol.) showed a decrease in the fluorescence lifetime of Trp214, τ_avg_ = 4.31 ns (A_1_ = 14%, τ_1_ = 1.44 ns, A_2_ = 86%, τ_2_ = 4.78 ns) in comparison to the neat albumin solution (before the addition of EtOH). The average fluorescence lifetime of Trp214 in HSA solution (native form) is 5.49 ns (A_1_ = 15%, τ_1_ = 1.65 ns, A_2_ = 85%, τ_2_ = 6.17 ns). Due to the fact that tryptophan in the ground state occurs in two isomeric forms, L-tryptophan and D-tryptophan (conformational isomers), different fluorescence lifetimes of this amino acid are observed in time-resolved measurements [36,37]. The lifetime of Trp fluorescence in proteins is approximately 2 and 6 ns, depending on the microenvironment in which it is located. The decrease in the Trp214 fluorescence lifetime and the shift of the maximum band in the HSA emission spectrum confirmed the structural changes in albumin in the presence of ethanol. However, analogous measurements were performed for HSA solutions (20 μM) containing 30% EtOH (vol.) and irradiated with an electron beam with different doses (0–7200 Gy) (Figure S7). Irradiation of these solutions with increasingly higher doses leads to a shorter fluorescence lifetime of Trp214 compared to the neat HSA solution. Our previous measurements show that the reaction of the hydrated electron with Trp induces a decrease in the fluorescence of Trp but does not cause any change in the fluorescence lifetime [31].

Valuable information was also obtained from the analysis of emission time measurements recorded at 420 nm (after excitation with a laser pulse of wavelength 337 nm) of BSA solutions (300 µM) saturated with N_2_O in the absence (blue line) and in the presence of 20% EtOH vol. (pink line). Both solutions were irradiated with a dose of 9000 Gy (Figure S8). Excitation of BSA solutions with 337 nm laser light selectively enables the detection of emission from excited protein aggregates, as this wavelength is not absorbed by monomeric albumin. BSA aggregates formed as a result of the irradiation of a solution containing ethanol are characterized by different emission decay kinetics recorded on an oscilloscope than the emission pattern of a BSA solution in which the aggregates were generated in the reaction with ^•^OH radicals (Figure S8). The emission decay at 420 nm is characterized by a two-exponential function with an average time of 3.37 ns in the case of ethanolic solution of BSA and 2.69 ns for the irradiated solution without EtOH (only the hydroxyl radicals are involved in the formation of aggregates). These measurements provide further evidence that the reaction of 1-hydroxyethyl radicals with protein causes the reduction in disulfide bridges and does not lead to the formation of dityrosine bridges (in the oxidation process).

## 3. Materials and Methods

### 3.1. The Sample Preparation

Essentially fatty acid-free albumin from human serum (HSA, A1887) and bovine serum albumin (BSA, A7030) were obtained from Sigma-Aldrich (St. Louis, MO, USA)/Merck (Darmstadt, Germany) and used as received. Protein solutions were prepared in ultrapure water containing 10 mM phosphate buffer (PBS, pH 7.2). Water was purified with the Hydrolab SPRING 20UV system. HSA, BSA, and ethanol were dissolved in phosphate buffer solution or in aqueous solution to appropriate concentrations immediately before measurements. Water–ethanol solutions of HSA or BSA were prepared in 10 mL volumetric flasks. To prepare them, we used one stock solution of HSA in water. The stock solution was kept overnight in the refrigerator and then filtered through a syringe filter (0.2 µm; Sartorius minisart syringe filter). In the next step, HSA stock solution (180 µM) and a small amount of water were added to the flask. The planned amount of ethanol was then slowly added, drop by drop, constantly stirring the contents of the flask. Finally, after waiting for the solution to reach room temperature, water was added up to the mark. The key to preparing the solution is to avoid elevated topical ethanol concentrations. This ensures slow addition of ethanol and vigorous mixing. Rapid addition of ethanol causes significant desolvation of albumin and formation of HSA aggregates, accompanied by turbulence. We always used 3 mL of solution for a single spectroscopic measurement.

The final concentrations of the solutions were verified spectrophotometrically (ε_280 nm_ = 35,500 M^−1^ cm^−1^ for HSA, ε_280 nm_ = 43,800 M^−1^ cm^−1^ for BSA). Concentrated albumin solutions foam strongly when saturated with gas, nitrogen, or nitrous oxide. Removing air from the solution using a vacuum system eliminates this problem. N_2_O saturation is challenging and often requires the use of a glove box. Albumin solutions containing ethanol foam much less when saturated with gases than those containing only protein. This may be beneficial when developing technological methods for obtaining albumin nanostructures using ionizing radiation. Additionally, in RLS measurements, each sample was filtered before measurement.

### 3.2. Optical Measurement

Steady-state emission spectra of the studied albumin solution were carried out using the Aminco-Bowman Series 2 (Hamamatsu R928, Hamamatsu Photonics, Shizuoka, Japan), and absorption spectra were recorded using a Perkin Elmer Lambda 750 spectrophotometer. Optical measurements are described in detail elsewhere [30]. In the measurements of stationary absorption spectra, we used optically matched cuvettes, and the reference solution contained an identical solution in which albumin was dissolved.

### 3.3. Pulse Radiolysis

Pulse radiolysis measurements were performed using a 6 MeV linear accelerator (LINAC) ELU–6E operating in single-pulse mode. The methodology and equipment for pulse radiolysis study with optical detection have been described in [30]. The solutions were saturated with ultrapure nitrogen, nitrous oxide, or were deaerated under vacuum. The samples subjected to electron beam irradiation also contained 0.1 M *tert*-butanol (*t*-BuOH) as a scavenger of ^•^OH radicals.

## 4. Conclusions

The aggregation process was studied using spectroscopic methods and pulse radiolysis, before and after irradiation of aqueous solutions of HSA or BSA containing different amounts of ethanol. The consequence of adding ethanol before irradiation of the protein solution was the formation of spontaneous HSA aggregates, but this occurs only with relatively high amounts of alcohol (above 40% vol.). For lower ethanol concentrations in the studied samples (below 40% vol.), no significant effect on the aggregation of HSA or BSA was observed. We did not observe significant changes in the shape of albumin fluorescence spectra and their intensity and lifetime in the range from 0% to approximately 30% ethanol (vol.). Also, in the case of RLS measurements, the spectrum of HSA changed only for the binary water/ethanol solution with a concentration of 60/40% by volume. The results of our RLS and NIR measurements are in good agreement and show that ethanol in albumin aqueous solution above 40% induced the formation of spontaneous protein aggregates.

The main aim of our research was to investigate the mechanism of the reaction of 1-hydroxyethyl radicals with albumins. We have shown that pulse radiolysis measurements are helpful for understanding the mechanism of reactions taking place in ethanol/water binary mixtures containing albumins. Pulse radiolysis of aqueous albumin solutions (30–90 µM) showed very high similarity when two alcohols, *t*-BuOH (0.1 M) or EtOH (5–30 vol%), were used as ^•^OH radical scavengers. Our experiments confirmed that CH_3_C^•^HOH radicals react with albumin via the same reductive pathway as hydrated electrons. Disulfide bridges in albumin molecules are the primary scavengers of reducing 1-hydroxyethyl radicals. Transient absorption spectra of albumins arise from the attachment of electrons to disulfide bonds, as previously described [5,31,38]. The reactivity of hydrated electrons with albumins is the same within aqueous solution (0.1 M *t*-BuOH) and water–ethanolic solution (contents EtOH below 40%). Moreover, the permanent changes induced by irradiation of the discussed solutions are similar, as shown by measurements of absorption, emission, and RLS spectra. It has been known for many years that CH_3_C^•^HOH radicals react with albumin, and differences can be expected when using EtOH and the non-reactive *t*-BuOH. 1-hydroxyethyl radicals are not very reactive in generating –S–S–, which is due to the redox potential and other channels of their decay. This is consistent with the observations of Schüssler’s earlier studies [11]. For high albumin concentrations (above 300 µM), the results of our pulse radiolysis measurements unequivocally prove that there is an electron transfer process to the cystine residues of the proteins. Another evidence for the reduction in disulfide bridges by radicals is the formation of a gel after irradiation of a solution (water/ethanol 70/30% vol.) containing 300 µM HSA saturated with N_2_O. Under the above experimental conditions, only ethanol radicals are formed, which induce the formation of aggregates (in this case, a gel) via interprotein disulfide bridges. The key methods for irradiating human or bovine albumin solutions to obtain hydrogels were described in our recent work [5]. As part of the present publication, we also obtained albumin hydrogels multiple times. The results of our research have shown that 1-hydroxyethyl radicals (solutions saturated with nitrous oxide) are able to initiate the formation of albumin nanostructures. This is consistent with the results of measurements using an electron beam for NA synthesis in a solution containing 20% ethanol (vol.) and in an atmosphere of nitrous oxide. An important difference in our measurements and those presented by [13] is the issue of the generation of dityrosine residue bridges. The results of our steady-state and time-resolved fluorescence measurements very clearly indicate the absence of tyrosine–tyrosine bridge formation. We believe that the emission spectra presented in the supplement to work [13] represent fluorescence spectra of albumin aggregates and not Tyr–Tyr bridges.

Radiolysis of binary water/ethanol solutions saturated with nitrous oxide or deprived of oxygen (vacuum or saturated with nitrogen) leads to the formation of only reducing radicals. Under these conditions, NA is formed as a result of the formation of intermolecular disulfide bridges–cystine residues.

We compared the results of our measurements of the radiolysis of albumin solutions with a relatively high ethanol content (10–40%) under reductive stress conditions with analogous results of studies on aqueous solutions of HSA or BSA with a low concentration of *tert*-butanol. The main conclusion from our measurements is the high similarity of the electron beam-induced processes in the case of using both of the above-mentioned alcohols. From the perspective of obtaining albumin nanomaterials, the use of ethanol at concentrations below approximately 40% does not provide any advantage over the “classical” irradiation conditions at a concentration of 0.1 M *t*-BuOH (0.1 M *t*-BuOH corresponds to a concentration of approximately 1% by volume). The use of excessive concentration of ethyl alcohol relative to water content (starting from twice the volume of EtOH relative to the volume of H_2_O and higher) to irradiated albumin solutions creates favorable conditions for the synthesis of interesting biomaterials. When there is an excess of ethanol relative to water in solutions containing albumin, we are dealing with classical desolvation, which leads to the formation of protein aggregates without the participation of covalent bonds. In the chemical synthesis of albumin aggregates by the desolvation method, –S–S– bridge reducing agent should be used to initiate the cross-linking process, leading to the formation of stable aggregates. In the radiation method, irradiation of solutions with effective albumin desolvation eliminates the use of chemical reducing agents. It simplifies the procedure for obtaining hydrogels and also ensures the sterilization of the final biomaterial.

We have shown that the use of ethanol concentrations below 40% does not provide any advantage over the classical hydroxyl radical scavenger *t*-BuOH (0.1 M). Only the use of a high concentration of ethanol in relation to water content (2:1) in irradiated albumin solutions creates favorable conditions for obtaining protein aggregates in the form of gels. For a wide range of readers involved in the synthesis of protein nanostructures by classical methods, the use of ionizing radiation for cross-linking without the use of chemical reductants should be interesting. This simplifies the procedure, and additionally, radiation sterilization of such products can be carried out simultaneously.

## Data Availability

Data are contained within the article and Supplementary Materials.

## Supplementary Materials

The following supporting information can be downloaded at: https://www.mdpi.com/article/10.3390/ijms26136283/s1.

## Abbreviations

The following abbreviations are used in this manuscript:

Ala	Alanine residue
AN	Albumin nanoparticles
BSA	Bovine serum albumin
Cys	Cysteine residue
EtOH	Ethanol, ethyl alcohol
HSA	Human serum albumin
NIR	Near infrared spectroscopy
RLS	Resonance light scattering
*t*-BuOH	*tert*-Butyl alcohol
