# Supplementary material for: Spectroscopic and Pulse Radiolysis Studies of Water–Ethanolic Solutions of Albumins: Insight into Serum Albumin Aggregation
Source: Int J Mol Sci. 2025 Jun 29;26(13):6283. doi: 10.3390/ijms26136283 (PMC12249838; doi:10.3390/ijms26136283)
Supplement: Supplementary file 1 [file ijms-26-06283-s001.zip › ijms-3701159-supplementary.pdf]

Supplementary Materials

Karolina Radomska, Marian Wolszczak*

*Correspondence: marian.wolszczak@p.lodz.pl

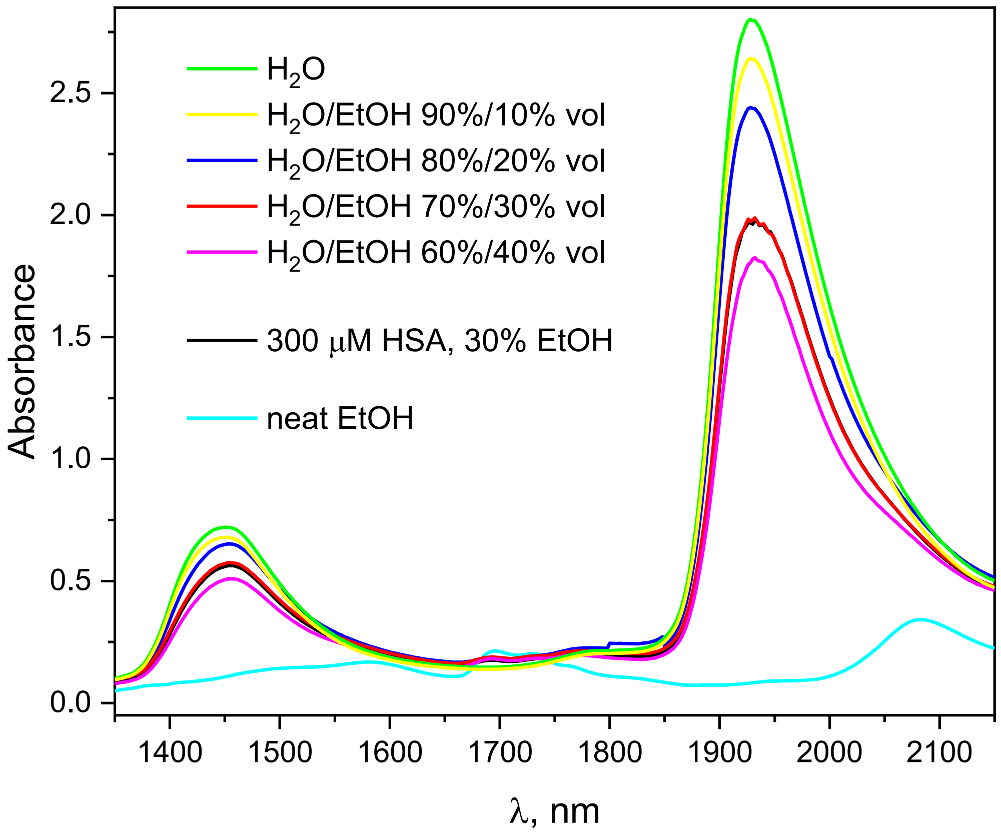

Figure S1. Absorption spectra of aqueous solution of EtOH (0-40% vol.). Absorption spectra of aqueous solution of HSA (300 μM) containing 30% EtOH (vol.) (black line). The cyan plot represents the spectra of neat EtOH. Measurements were made using a 0.5 mm quartz cuvette.

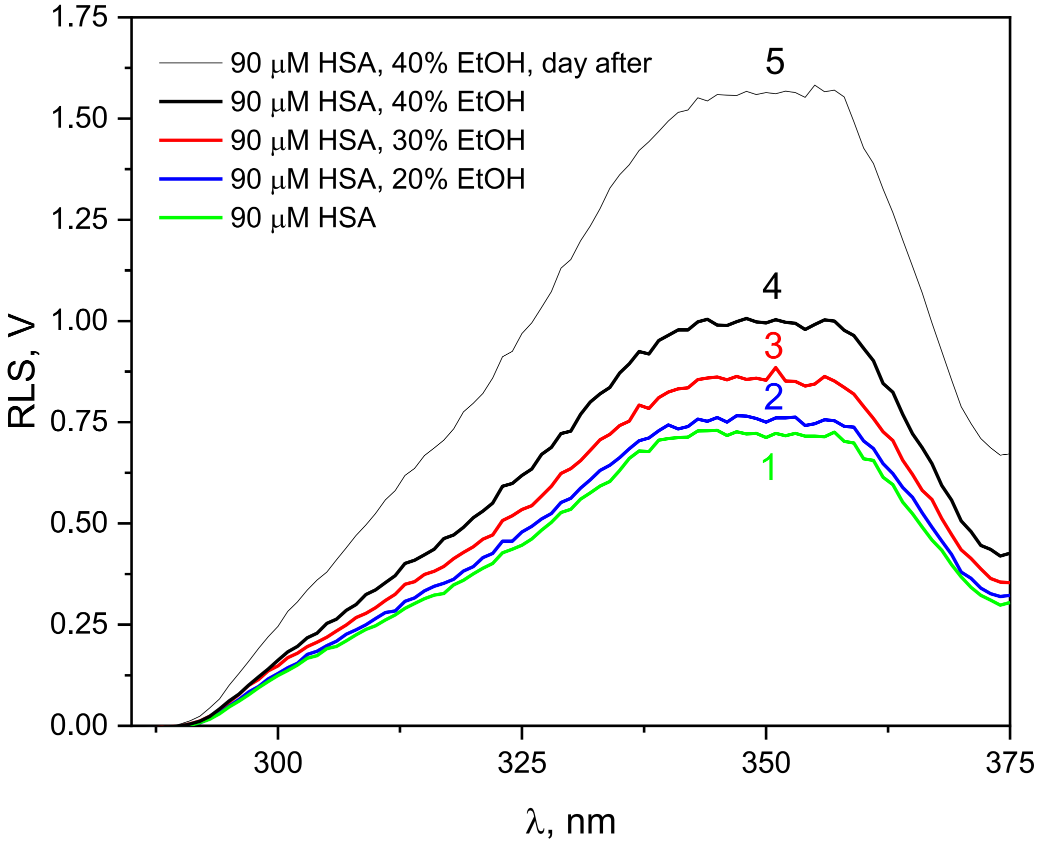

Figure S2. RLS spectra of 90 μM BSA: (1) aqueous solution, (2) aqueous solution containing 20 % EtOH, (3) aqueous solution containing 30 % EtOH, (4) aqueous solution containing 40 % EtOH, (5) aqueous solution containing 40 % EtOH recorded the day after. The spectra recorded one day later for BSA solutions without ethanol (1) and containing 20% (2) or 30% (3) do not change in the slightest. In the case of BSA aqueous solution containing 40% EtOH, a large increase in the RLS signal intensity was observed (curve (5)) the next day.

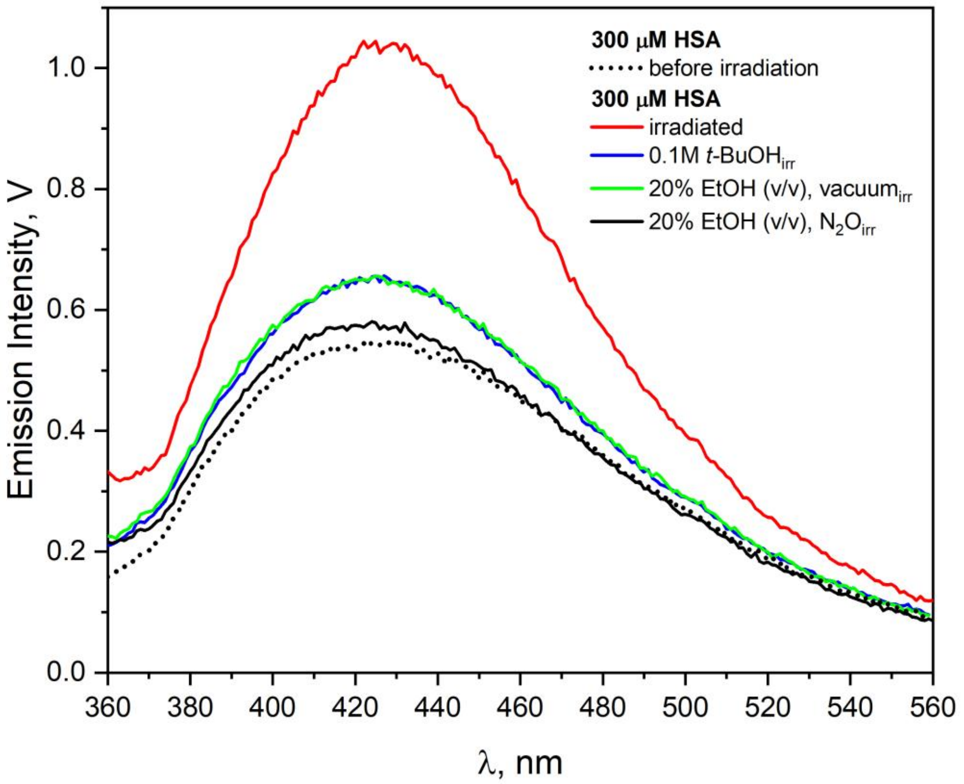

Figure S3. Emission spectra of the HSA solutions (300 μM) before (black dotted line) and after irradiation with dose 9000 Gy: containing 0.1M *t*-BuOH (blue line); containing 20% EtOH (green line) or containing 20% EtOH and N_2O saturated (black line). Emission spectrum of the N_2O-saturated HSA solution (300 μM) after irradiation with dose 9000 Gy represents red line. The excitation wavelength was 337 nm.

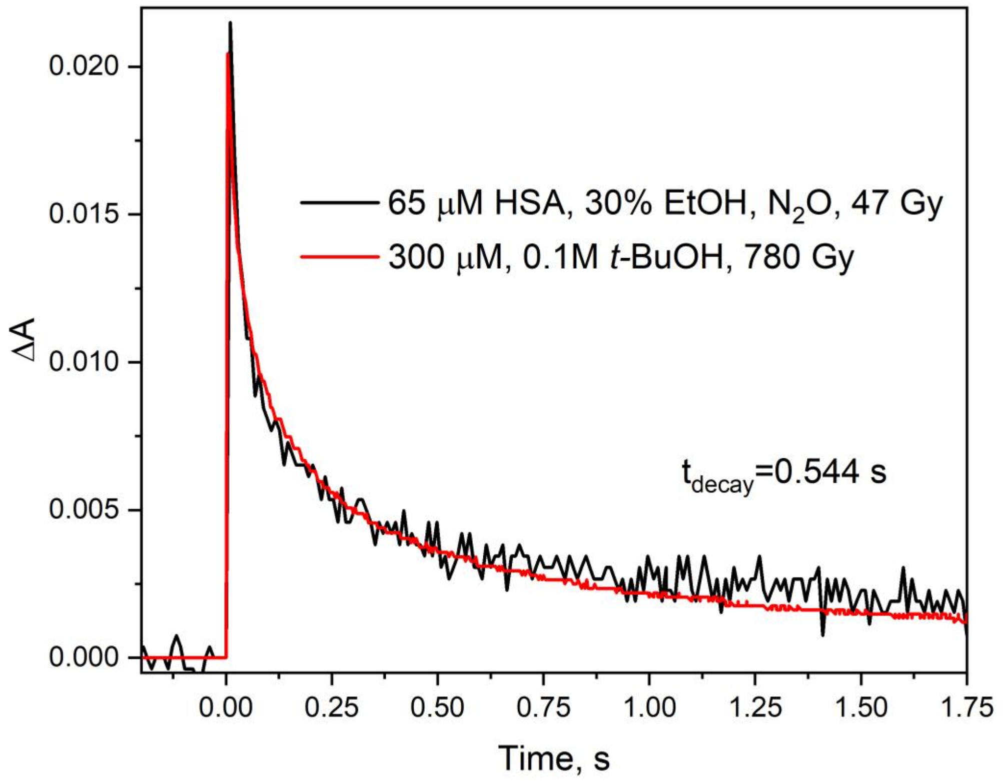

Figure S4. Time profiles of the absorbance recorded at 420 nm after 17 ns pulse irradiation with a dose of 47 Gy of the vacuum deaerated aqueous solution containing HSA (65 μM) and *t*-BuOH (0.1M). Time profiles of the absorbance recorded at 420 nm after 17 ns pulse irradiation with a dose of 780 Gy of the N_2O-saturated aqueous solution containing HSA (300 μM) and EtOH (30% vol.). t_{decay}=0.54 s in the case of HSA solution without ethanol and HSA solution containing ethanol. The kinetics are normalized.

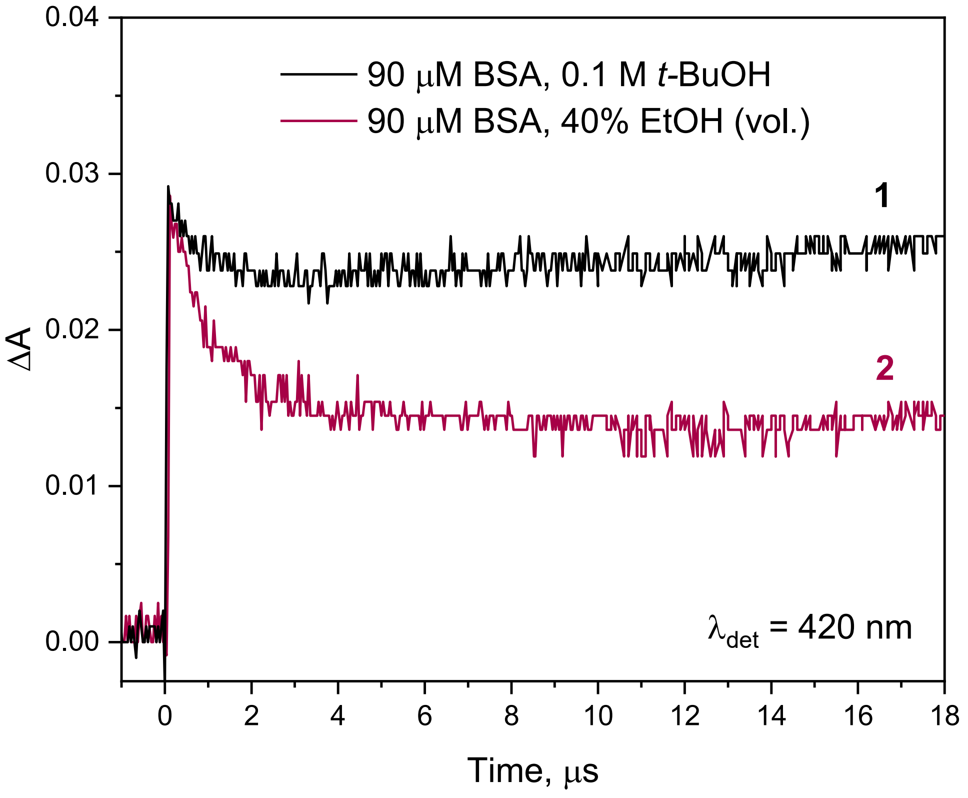

Figure S5. Time profiles of the absorbance recorded at 420 nm after 17 ns pulse irradiation with a dose of 33.9 Gy of the vacuum deaerated aqueous solutions: containing BSA (90 μM) and *t*-BuOH (0.1M) (curve 1) or BSA (90 μM) and EtOH (40% vol. curve 2).

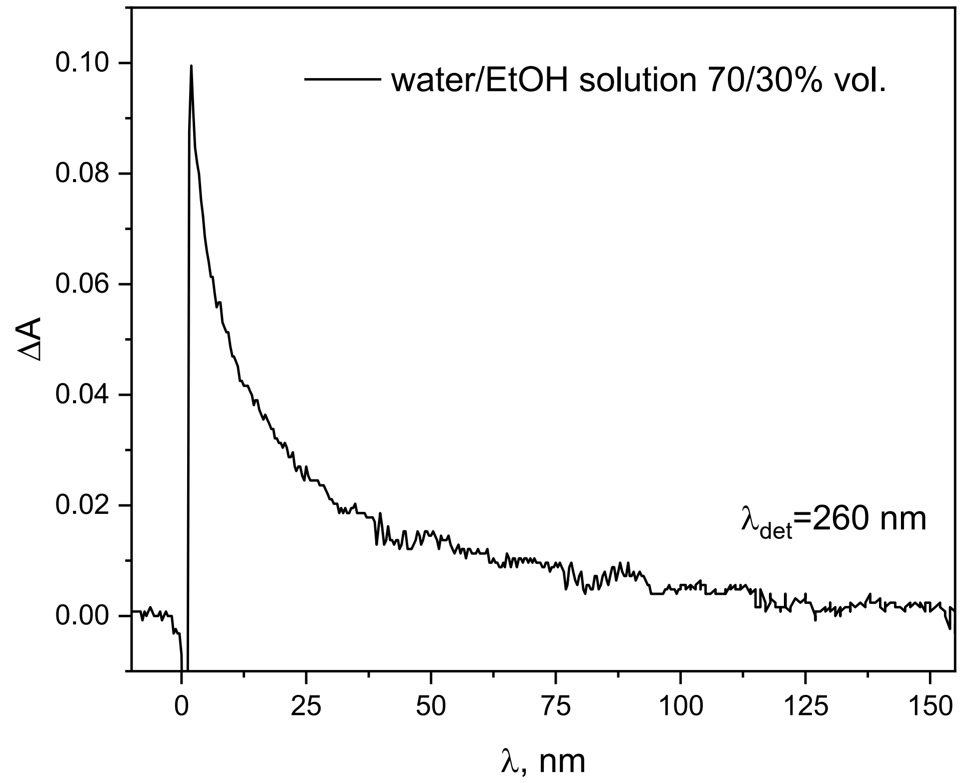

Figure S6. Time profiles of the absorbance recorded at 260 nm after 1 μs pulse irradiation with a dose of 200 Gy of the vacuum deaerated aqueous solutions containing 30% EtOH (vol.).

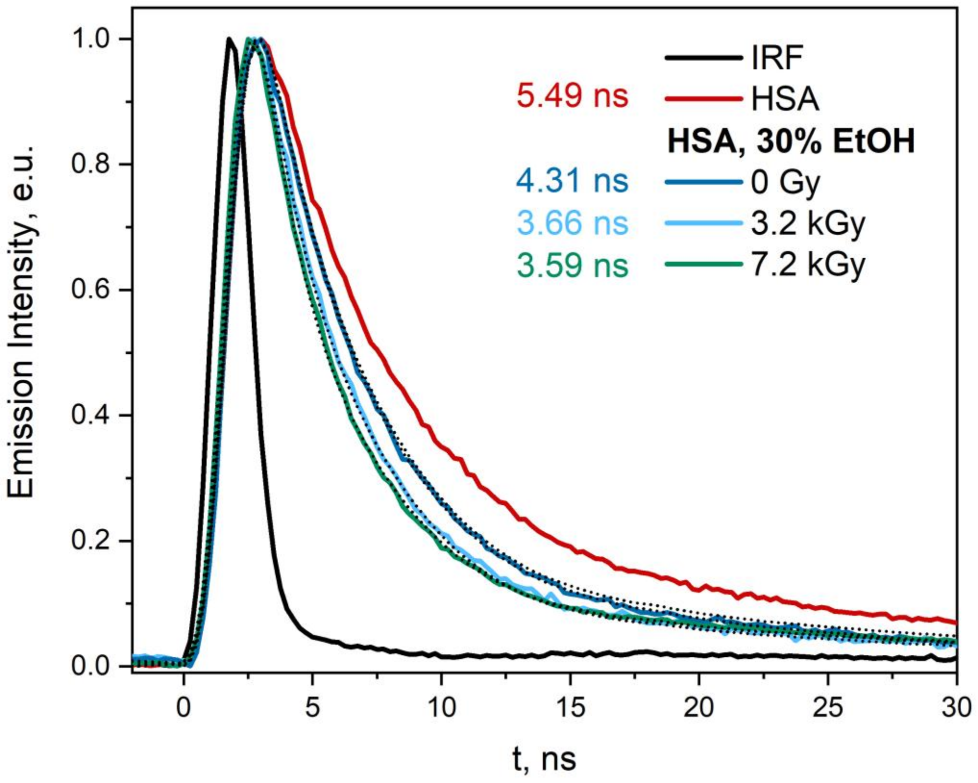

Figure S7. Decays of fluorescence of the neat HSA solution (20 μM) and solutions of HSA (20 μM) containing 30% EtOH (vol.) after irradiation with dose 0–7200 Gy. The curves were normalized to I=1.

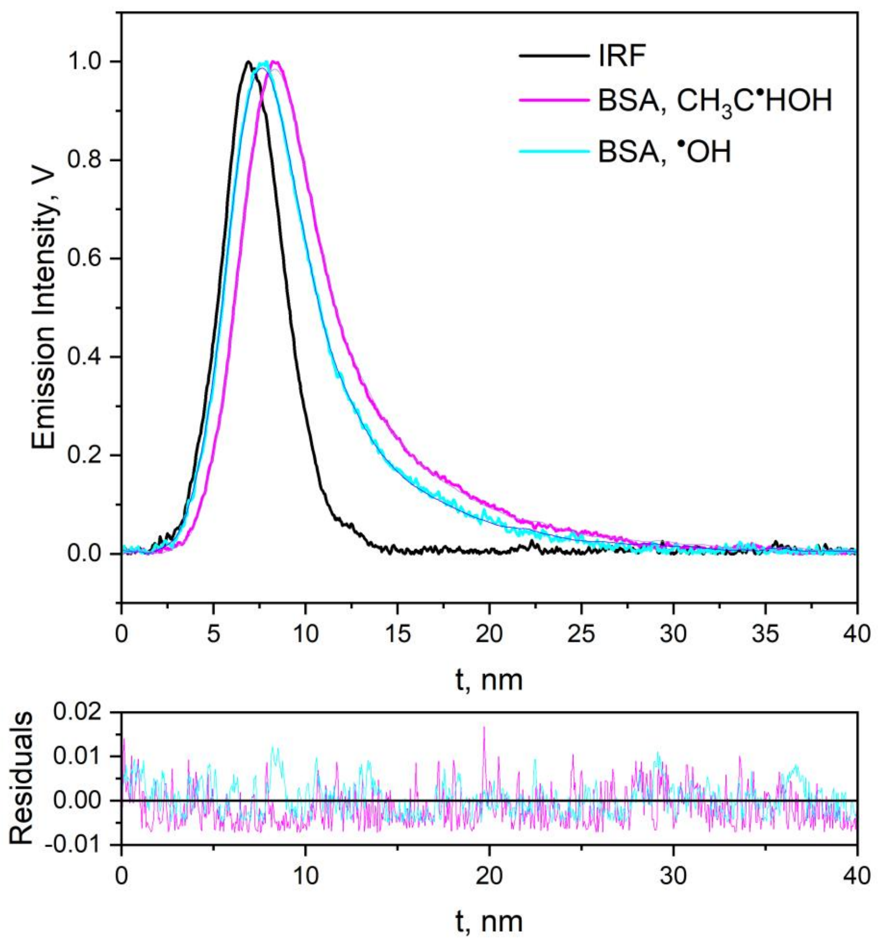

Figure S8. Decays of the emission of BSA solution (300 μM) containing EtOH (20% vol.; pink line) and solution of BSA (300 μM; blue line). Both solutions were saturated with N_2O and irradiated with dose 9000 Gy. The smooth lines represent biexponential fits to experimental curves (noisy ones). The solutions were excited with nitrogen laser pulse (337 nm) and emission was analyzed at 420 nm.
